# Senescent Microvesicles: A Novel Advance in Molecular Mechanisms of Atherosclerotic Calcification

**DOI:** 10.3390/ijms19072003

**Published:** 2018-07-09

**Authors:** Matilde Alique, Rafael Ramírez-Carracedo, Guillermo Bodega, Julia Carracedo, Rafael Ramírez

**Affiliations:** 1 Biology Systems Department, Physiology, Alcala University, Alcala de Henares, 28805 Madrid, Spain; manuel.ramirez@uah.es; 2 Cardiovascular Joint Research Unit, University Francisco de Vitoria/University Hospital Ramon y Cajal Research Unit (IRYCIS), 28223 Madrid, Spain; rrcarracedo@hotmail.com; 3 Biomedicine and Biotechnology Department, Alcala University, Alcala de Henares, 28805 Madrid, Spain; guillermo.bodega@uah.es; 4 Department of Genetic, Physiology and Microbiology, Faculty of Biology, Complutense University/Instituto de Investigación Sanitaria Hospital 12 de Octubre (i+12), 28040 Madrid, Spain; julcar01@ucm.es

**Keywords:** atherosclerosis, microvesicles, senescence, endothelial senescence, vascular calcification, aging

## Abstract

Atherosclerosis, a chronic inflammatory disease that causes the most heart attacks and strokes in humans, is the leading cause of death in the developing world; its principal clinical manifestation is coronary artery disease. The development of atherosclerosis is attributed to the aging process itself (biological aging) and is also associated with the development of chronic diseases (premature aging). Both aging processes produce an increase in risk factors such as oxidative stress, endothelial dysfunction and proinflammatory cytokines (oxi-inflamm-aging) that might generate endothelial senescence associated with damage in the vascular system. Cellular senescence increases microvesicle release as carriers of molecular information, which contributes to the development and calcification of atherosclerotic plaque, as a final step in advanced atherosclerotic plaque formation. Consequently, this review aims to summarize the information gleaned to date from studies investigating how the senescent extracellular vesicles, by delivering biological signalling, contribute to atherosclerotic calcification.

## 1. Introduction

Atherosclerosis is a chronic inflammatory process closely related to aging that causes the narrowing of the arterial lumen due to the development of plaque in the tunica intima of the arterial wall. The atherosclerotic plaque (AP) might develop silently over decades and predisposes the individual to cardiovascular disorders. Atherosclerosis is a prevalent problem in developed countries and is the most frequent cause of death worldwide attributed to cardiovascular diseases (CVDs). Its main features are endothelial dysfunction, intimal thickening, inflammation and vascular calcification (VC) [1,2,3]. To date, oxidative stress, endothelial dysfunction and inflammation play a critical role in the atherosclerotic plaque progression [4]. Aging is related with increased incidence, prevalence and mortality associated with atherosclerosis [5]. Generally, the atherosclerosis stages are well known but one of the most important final steps in atherosclerosis progression is the calcium mineral deposits in the vascular area of advanced (also named complicated) plaques [6,7]. Moreover, cardiovascular calcification is a growing burden in aging societies of occidental descent and contributes to cardiovascular disease.

Extracellular vesicles (EVs) are a heterogeneous group of membranous subcellular structures secreted to the extracellular compartment. EVs are different in size, biogenesis and release and are usually classified into three categories: exosomes, microvesicles (MVs)—also known as microparticles, ectosomes or matrix vesicles—and apoptotic bodies [8,9]. They are produced by all the cellular types, especially if cells are in contact with liquids like synovia, milk, urine or blood and the MVs could release to body fluids or interstitial fluids. The cargo of EVs consists of a variety of molecules including bioactive proteins, lipids, DNA, RNA and microRNAs (miRNAs). MVs are released from the cell by the budding of the plasma membrane and represent not only a heterogeneous structural population since their size usually ranges approximately from 0.15 to 1 μm in diameter. More specifically, plasma MVs also represent an abundant and heterogeneous group of MVs. Their heterogeneity is due to their ability to be produced by erythrocytes, leucocytes, platelets and endothelial cells (ECs). Data of plasma MVs quantification are uneven, although an accepted value of total plasma MVs could range from 200,000 to 400,000 MVs/μL [10].

Plasma MVs have been involved in the regular maintenance of ECs [11] and other blood-related physiological functions such as coagulation, reticulocyte maturation and angiogenesis [9]. EVs between them, exosomes and MVs, also have a significant role in senescence, where they are increasing more than 10-fold in most cases [12,13,14]. The pathogenesis of different diseases [15] including cardiovascular pathology [16], is a reason why they also have been used as therapeutic tools in cardiovascular disease [17]; more specifically, plasma MVs are mediators of endothelial dysfunction [18] and atherosclerosis development [19,20,21,22] including calcification [23].

Cellular senescence and especially endothelial senescence is a common biological effect in different CVDs generated by some causes such as aging, hyperlipidaemia, diabetes mellitus, hypertension (HT), smoking and uremic toxins, among others. Another common biological effect observed in senescent cells is their higher production of MVs [12,13]. Interestingly, the involvement of MVs in the has been demonstrated with the most abundant evidence [6,24,25,26,27,28], therefore, this review is focused on the possible role of the MVs in different CVDs and more precisely in the development and calcification of AP while paying as particular attention as possible to the role of senescent MVs.

## 2. Atherosclerosis

Atherosclerosis is a chronic pathology with multifactorial causes. The development of atherosclerosis follows a multi-step and complicated process during which interactions in the cause-effect relationship are often difficult to distinguish. What is clear is that in the atherosclerosis development is implied the “atherosclerosis triad”: (1) oxidative stress (reactive species, such as reactive oxygen species (ROS) and reactive nitrogen species (RNS)), (2) endothelial dysfunction and (3) inflammation [4]. More specifically, in human atherosclerotic lesions are implicated many cytokines, adhesion molecules (vascular cell adhesion molecule-1 (VCAM-1), intercellular adhesion molecule-1 (ICAM-1) and L-selectin) [29], metalloproteinases (MMPs) [30,31,32,33] vasodilatory factors (nitric oxide (NO) and prostacyclin) and promoting a pro-adhesive and pro-thrombotic phenotype [30,33,34,35,36].

The development of atherosclerosis follows the same course. First, ECs are damaged (endothelial dysfunction) by altered hemodynamic forces where blood flow is low and disturbed contributing to the recruitment of inflammatory cells. Accumulating evidence suggest that endothelial dysfunction is an early marker for atherosclerosis [37], therefore, the discovery of new mediators, such as MVs, as an early marker in the atherosclerotic process could be a therapeutic target. ECs take native low-density lipoproteins (nLDL) due to changes in endothelium permeability at sites of lesions in specific areas and then into the vessel wall—nLDL undergo oxidation by oxidative stress (ROS and RNS). These atherogenic stimuli—oxidized low-density lipoproteins (oxLDL)—can then activate the inflammatory response and stimulate the overlying ECs to release miRNAs, growth factors and cytokines (molecules implied in adhesion and chemotaxis processes, growth factors and a reduction of NO production) [37,38,39]. The consequence of the activation of the inflammatory response is the recruitment of monocytes and macrophages, which interact with highly oxLDL to form foam cells. Additionally, the upregulation of proinflammatory cytokines produces a vascular smooth muscle cell proliferation. The last step is plaque formation or atheroma when intimal smooth muscle cells produce an extracellular matrix that gives rise to a fibrous cap characterized by its susceptibility to rupture and thrombus formation, subsequently leading to an acute vascular occlusion [40,41,42,43]. Calcifications in the thin AP can contribute to plaque destabilization [44] and subsequent rupture, leading to myocardial infarction and stroke. These arterial-wall-specific mechanisms have been corroborated by recent human genetic evidence [45,46].

Generally, during atherosclerosis progression also occur alteration in mineral metabolism, modification of calcification regulators and changes in vascular cell phenotype [47]. Intimal calcification has been reported in the first steps during the development of advanced atherosclerotic lesions; the initial point is the intimal permeability that is increased in low-level atherosclerosis and as a consequence is developed an advanced atherosclerotic lesions over time and atherosclerotic intimal calcification [48]. Results reported that in the development of advanced atherosclerotic lesion contributes the intimal calcification itself and the determination of calcium score is a well-known marker in the VC [48]. Recent evidence on the study of mineralization reports that in the MVs from ECs [49], smooth muscle cells and macrophages play a critical role as mediators in the development of APs and subsequently calcification [27].

Genetic evidence is supporting arterial calcification are linked to atherosclerosis. A large scale of genomic studies showed substantial overlapping of risk loci between coronary arterial calcification produced by smoking [50], coronary artery disease [51], myocardial infarction [51,52], atherosclerosis [53], ethnic-specific [54] and diabetes mellitus [55], indicating shared pathological components.

## 3. Senescence

Senescence is defined as process that occurs in all biological systems and produced the continuous deterioration of structures as well as functions of cells, tissues, organs and organ systems [56] and is recognized as the state of irreversible cell cycle arrest [57]. The process by which cells achieve senescent status is due to the cell aging itself (biological aging), breaking homeostasis and promoting chronic age-related diseases (premature aging). Some disorders are more prevalent with aging, such as atherosclerosis, dementia, diabetes, blindness, chronic kidney diseases (CKDs) [58], osteoarthritis among others and used to be linked with elderly people [59,60]. Moreover, cell senescence processes appear to be involved in physiological processes of control such as cancer protection, biological developmental processes, tissue repair in aging situations and age-related disorders [61].

Cellular senescence can be considered a programmed change in cell state that occurs in two steps: cell cycle arrest and gerogenic conversion (geroconversion). Geroconversion is a form of growth that converts reversible arrest to irreversible senescence. Moreover, this process is often accompanied by conversion to hyper-secretory, pro-inflammatory and immunogenic phenotypes and cellular malfunctions [62]. Geroconversion on organismal level leads to age-related diseases and death. Accordingly, the aging cell is a damage consequence through the time due to an accumulation of mistakes in the regular cell function. For this reason, senescence appears as a final step of fails in the cell machinery which are or not involved the aging, therefore, independent of age [62].

According to the mechanism of induction, senescence could be classified in at least three types: (1) senescence-associated secretory phenotype (SASP), such as replicative senescence model in vitro or aging in vivo, (2) stress-induced premature senescence (SIPS), such as ionizing radiation can induce and (3) oncogene-induced senescence (OIS). SASP is a phenotype linked to telomerase activity diminished and telomere shortening [63], whereas SIPS is not associated with telomeres, thus it is considered as a reversible phenomenon [64]. Regarding OIS, the proliferative arrest involves changes in the activation of different oncogenes, that is, pRB (retinoblastoma protein), Ras protein or tumor protein p53 [65,66].

Both processes, SASP (naturally aged cells) and SIPS (prematurely aged cells), present common characteristics between them, markers of senescence, proliferation changes, DNA damage, increased the sensitivity to cell death and cell dysfunction. Most of these features are detected in cells in the AP [67,68].

During atherosclerosis progression, ECs are subjected to damaging stimuli (growth factors and proinflammatory cytokines) and the consequence of the long-term cellular response to this stress is the activation and development of the process of endothelial senescence. Data are scarce about the possible link between MVs and senescent process [49,69], although it is well known that senescent cells produce a higher number and different MVs than young cells [49,70].

Senolytic drugs, recently discovered, selectively destroy senescent cells by causing apoptosis [71,72,73] and making senescent cells susceptible to their own pro-apoptotic microenvironment [60]. This connection highlights the senescent cells resistant to apoptosis implying these cells present all the mechanism pro-survival elevated. Indeed, these senescent cells are characterized by anti-apoptotic pathways (SCAPs) decreased which might be a compensatory mechanism related to senescence-associated mitochondrial dysfunction (SAMD). SAMD is a type of SASP-related senescence due to mitochondrial membrane potential deregulation. Senescent cells develop a resistant to death through of up-regulated survival factors that may explain their sensitivity to SCAP-focused treatments compared with non-senescent cells [60]. Consequently, senolytics and SASP inhibitors open a new therapeutic approach in elderly subjects and patients with chronic diseases that could benefit and enhance their lifespan and therefore, their healthspan.

Some senescent cellular markers are described in Table 1. Most of them are involved in DNA repair and cycle control [74]. Generally, regarding DNA regulation, senescent cells decline in DNA replication, suffer a reorganization of chromatin into discrete foci and are associated with histone modifications (senescence-associated heterochromatin foci; SAHFs) and the overexpression of different DNA repair proteins (senescence-associated DNA damage foci; SDF). Between the SDF, there are two important markers that increase, γ-H2AX (phosphorylated histone H2AX), a marker of DNA double-strand breaks and genomic instability and 53BP1 (p53-binding protein-1), a protein associated with DNA damage. Moreover, other markers have been highlighted as a common senescent cellular marker, like senescence-associated-β-galactosidase (SA-β-gal) and p16 [65]. SA­β-gal derives from the lysosomal β­galactosidase and reflects increased lysosomal biogenesis. Recently, SA-β-gal and p16 are considered as a not robust marker because they tend to overestimate the number of positive senescent cells. Therefore, new markers of senescence are described, among them cyclin D1 and lamin B1 [75,76] and are considered more reliable and reproducible. The proteins that play a role in the cycle control are early markers of DNA damage-induced senescence.

### 3.1. SASP

The next sections (Section 3.1.1 and Section 3.1.2) will show, in the case of SASP, cellular senescence is not only due to telomeres shortening; aging factors such as ROS and inflammatory molecules also contribute to the irreversible loss of replicative capacity [84].

#### 3.1.1. Replicative Senescence

Hayflick and Moorehead [85] demonstrated in 1961 that human fibroblasts could proliferate vigorously for dozens of generations following serial subcultivations but after approximately 50–70 generations the cells' growth arrested. This in vitro phenomenon has been termed “replicative senescence.” Replicative senescence is defined as a phenomenon in which cell divided multiple times losing their replication capacity through serial passages in culture, as a result, senescent cells express specific biomarkers (see Table 1). The involvement of replicative senescence in the aging process was postulated by Shay and Wright [63] with the term Hayflick limit: the number of times that a normal cell in culture will divide until cell division stops. The reason why most primary cells have a limitation in their replication is the telomere shortening produces each new cell division until they shorten to a critical length [63]. Primary culture cells are characterized by their low telomerase expression, then every time that they are divided, their telomeres suffer attrition [86].

#### 3.1.2. Aging

Aging is a degenerative process caused by accumulated damage that leads to cellular dysfunction, cellular senescence, tissue failure and death. Biological aging is a cell aging process which, by itself, triggers homeostasis malfunctions and subsequently, degeneration of cell functions and, in general, in all organs and tissues malfunctions increasing risk of age-associated disease, between them age-related neurodegenerative diseases [87] and atherosclerosis [88]. Note that aging always involves senescence but senescence does not necessarily involve aging, however, senescence has also been implicated as a major cause of age-related disease [89].

The theory of oxidation-inflammation has been proposed as the primary cause of aging (oxi-inflamm-aging) which described a strictly relationship between oxidative stress levels and immune cells function in the longevity of individuals [73,90]. In agreement with the oxidation-inflammation theory of aging, the base of immunosenescence is the age-related chronic oxidative and inflammatory stress of the immune cells [90,91]. In fact, heat shock protein 70 (Hsp70) has been described as an elemental chaperone that participate in the oxidative stress level maintained and in the inflammatory response modulation, both playing a role in the aging process [91]. Recently, it has been demonstrated that EVs participate in these processes due to play a role in the immune response as a result of EVs contained lipids [92].

It is well known that the mitochondria are sensitive to oxidative stress and therefore, plays a critical role in the theory of aging [87,93], a fact recently termed Mitochondrial Free Radical Theory of Aging (MFRTA) [94]. According to this theory, a step beyond the free radical theory of aging described until now, the oxidative damage of mitochondrial DNA generates mitochondrial dysfunction, although other possible mechanisms can also be involved in this mitochondrial impairment [95,96]. Cells from different tissues are continuously exposed to oxidative stress and pro-inflammatory factors. Exogenous nitrate administration increased ROS/RNS stress in the elderly [89] for example. Regarding the reactive species stimuli are constant over time and along of the lifespan. Oxidative stress affects some targets, between them proteins, lipids and DNA [97]. ROS/RNS stress produces polyunsaturated free fatty acids oxidation and generate LDL oxidation, changing the cell membrane characteristics (fluidity and permeability) and also as atherogenic stimuli develop CVDs such as atherosclerosis [98].

Most recently, some studies demonstrated that cell senescence promotes biological aging as well as age-related diseases [61,99,100]. Moreover, the relationship between senescence, mitochondrial damage (previously described in MFRTA) and lipid oxidation (as a consequence of oxidative stress) open a new field of discussion [98].

### 3.2. SIPS

**Figure 1:**
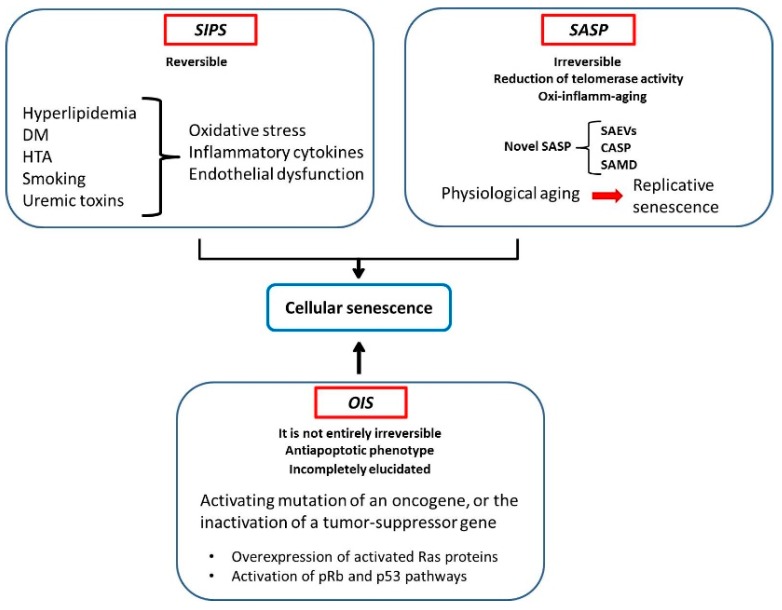
Types of senescence. Stress-induced premature senescence (SIPS), senescence-associated secretory phenotype (SASP) and, oncogene-induced senescence (OIS). Senescence-associated EVs (SAEVs); CKDs-associated secretory phenotype (CASP); senescence-associated mitochondrial dysfunction (SAMD).

Since the birth, all biological systems have been exposed to stress due to environmental factors and their own metabolism. The immediate cellular response to stress is the activation of detoxification mechanisms. The primary cause of SIPS is due to an accumulation of a long-term cellular response to stress when the organism is incapable of detoxifying this stress and/or eliminating damage stimuli [64]. The net result of the damage generated by stress and the efficiency of the stress response is unbalanced in this case. A theoretical framework that helps to explain the relationships between stress and senescence is included in Figure 1.

To date, some chronic pathologies have been associated with a premature aging process and considered as SIPS. Some of them are hyperlipidaemia, diabetes mellitus, HT, smoking and uremic toxins. It is interesting to remember that almost thirty years ago, Dzau [101] described how some associated-pathologies like HT, dyslipidaemia and glucose intolerance, cluster in the population and act synergistically in increasing and interacting with atherosclerosis.

#### 3.2.1. Hyperlipidaemia

A large number of evidence shows that hyperlipidaemia, the excess of lipids, is a risk factor for almost all the cardiovascular diseases which are the leading cause of death in the population of western countries [5].

Regarding hyperlipidaemia, the oxLDL seems to be the most important causal agent. Although, it has been suggested recently that long-term treatment of nLDL could also induce the premature senescence of ECs [102]. ECs treated with nLDL in vitro show proliferation inhibition in this study and achieve a senescent phenotype, probably due to the production of ROS. These data suggest a negligible contribution of oxLDL to the in vivo development of atherosclerosis and cellular senescence in the absence of pathological conditions, such as hyperlipidaemia or diabetes mellitus [102].

OxLDL is produced when nLDL is scarce and irreversibly oxidized due to active circulating oxidants [103]. OxLDL causes multiple cardiovascular diseases [104,105], playing a role in age-related vascular pathologies such as stroke, coronary heart disease (CHD) and atherosclerosis, probably because oxLDL exerts damage effects on vascular ECs (endothelial dysfunction). The NO synthase uncoupling generate endothelial nitric oxide dysregulation [106], adhesion molecules modulation in the ECs, [107], vascular oxidative stress induction [108] and induction of apoptosis [109] and senescence [110] of ECs are effects caused by oxLDL. More specifically, the senescent status induced by oxLDL is characterized by a cell-cycle arrest and pro-inflammatory changes in gene expression [30] and several studies have revealed that senescent ECs are present in human atherosclerotic plaque [35,111,112].

It is well known that atherosclerosis, the hallmark sign of CVDs, is caused by dyslipidaemia and some studies have shown the role of EVs in the different stages of atherosclerosis [7,20,21,22]. Moreover, the number and phenotype of MVs have been associated with major cardiovascular risk factors such as smoking, diabetes mellitus, obesity, HT, dyslipidaemia and metabolic syndrome [19].

#### 3.2.2. Diabetes Mellitus

Diabetes mellitus is a risk factor for several pathologies highlighted CVDs (CHD, ischemic stroke and peripheral arterial occlusion), CKDs, neuropathy and retinopathy (microvascular disease) [113]. Concretely, diabetes mellitus is associated with high rates vascular diseases (coronary artery disease, cerebral vascular disease and peripheral arterial disease) [42]. In the origins of diabetic atherosclerosis, not only the direct effects of chronic hyperglycaemia are implied *per se*, insulin resistance, non-esterified free fatty acid (NEFA) production, dyslipidaemia, hypercoagulability and impaired response to injury also contribute to this process [41,42,43]. The cells affected by diabetes mellitus on the vasculature are extensive but the principal cells are endothelium and smooth muscle cells [41].

Plenty of data exists showing that diabetes mellitus patients have accelerated atherosclerotic vascular disease [114]. Moreover, evidence indicates that diabetes accelerates the process of aging and endothelial cell senescence, in particular in high-risk patients who develop complications [115]. The endothelial senescence is associated with some diabetes vascular complications highlighting diabetes retinopathy, which is one of the most common complications of diabetes.

Hyperglycaemia generates increased levels of advanced glycosylation end products (AGEs) and, as a consequence, an acceleration of atherosclerosis due to the non-enzymatic reaction between glucose and proteins and/or lipoproteins in arterial walls and oxidative stress [116]. A recent study described EVs as a novel effector that implied the interrelation risk factors in a cluster of cardiovascular disease and diabetes mellitus (metabolic syndrome) [117]. To conclude, EVs in diabetes mellitus, together with pathological processes including aging and inflammation, both are implied in oxidative stress-induced vascular cell senescence [16,118,119].

#### 3.2.3. Hypertension

Together with atherosclerosis, hypertension (HT) is described as one of the main health problems in the developing countries due to its high prevalence and its association with others diseases [120]. The first evidence which described the role of HT in atherosclerosis dates from 1976. Hollander [121] demonstrated that HT, currently termed high blood pressure (HBP), is a major factor in CHD, stroke, renal disease, peripheral vascular disease and other disorders. HT and the resulting increase in the blood pressure on the myocardial and arterial walls lead to the development of hypertensive heart disease. HT generates shear stress on the arterial wall and produces the acceleration of atherosclerosis [101]. HT patients, experiencing atherosclerosis or only HT, are progressing to occlusive disease of both the large and small arteries which results in myocardial infarction, stroke and other diseases aforementioned.

The endothelium is the central focus of the effect of both diseases atherosclerosis and HT. HT causes endothelial injury and the increased pressure and/or vasoactive substances produces vascular cell proliferation, both results contribute to the atherosclerosis progression and acceleration of this process. Additionally, diabetes and hyperinsulinemia also generate some effects in the vasculature, increasing vascular tone and vascular smooth muscle cell proliferation and endothelial dysfunction [101]. It is well established in a somewhat parallel fashion, that HT is associated with increased oxidative stress and induces the premature senescence of ECs in the arterial wall [122]. However, there was a debate whether oxidative stress was a cause or a consequence of HT. Some authors have demonstrated in clinical studies that antioxidant supplements have failed in the regulation of blood pressure, whereas it was noteworthy that antihypertensive treatment reduced blood pressure was associated with a decrease in oxidative stress [123]. Conversely, other results have been published regarding how the supplementation of antioxidants reduces blood pressure, supporting that oxidative stress is the cause of HT [124].

Many studies have associated HT with a change in the number and characteristics of MVs, mainly those produced by platelets or endothelium [125]. Additionally, it has been proposed that the release of MVs associated with HT can be modulated by means of treatments, constituting an interesting therapeutic objective [126].

#### 3.2.4. Smoking

Cigarette smoking is the primary cause of preventable morbidity and mortality in the Western world. Smoking, with the previously described factors, is a well-established cardiovascular risk and generates endothelial damage during all the phases of atherosclerosis, the latter being mostly thrombotic [127]. Recently, it was identified as a significant risk factor in the progression of CKDs [128]. Several compounds in cigarette smoke, such as nicotine, have been demonstrated to alter endothelial function and therefore, accelerate atherosclerosis progression [128].

The continuous exposition to cigarette smoke produces effects in the endothelium, the majority of them are created by reactive species: ROS and RNS. Reactive species contribute to oxidative stress in this regard, via upregulation of inflammatory cytokines, endothelial dysfunction and as the last step of endothelial damage, the induction of the premature senescence of ECs [129]. Thus, the smoking exposition affects in all stages of atherosclerotic plaque formation and development [130]. Interestingly, some studies suggest that the principal compound of cigarette, nicotine, produces oxidative stress increase that induces extracellular matrix production in human mesangial cells, thus showing the smoking effects in renal disease [128].

Recently, a study reported a novel pathogenic mechanism for airway remodelling in chronic obstructive pulmonary disease, which can be related to smoking habits stress-induced MVs from bronchial epithelial cells [131]. Moreover, cigarette smoke extract results in the release of pro-inflammatory MVs in a concentration-dependent manner [132]. Plasma EVs levels and their content might be affected by smoking in healthy subjects [133]. It seems logical to think that these changes in MVs can be responsible for modifications involved in the atherosclerosis processes.

#### 3.2.5. Uremic Toxins

There is a wealth of data evidencing the intimate links between CKDs and CVDs [134,135,136]. Thus, uremic toxins, considered as a non-traditional risk factor in CVDs, play a crucial role in the pathogenesis of atherosclerosis in patients with CKDs [137,138]. Specifically, atherosclerosis is an inflammatory vascular disease frequently associated with renal disease due to endothelial dysfunction [139]. Indeed, some studies have proven that one of the atherosclerotic processes by which the CVDs are developed is endothelial senescence [35,140].

The European Uremic Toxin Work Group (EUTOX) listed at least 88 uremic toxins in CKDs [141]. Nevertheless, despite the development of new dialysis techniques for patients with renal dysfunction, in spite of that, the size of some uremic toxins are larger than the pore size of dialysis membranes and are poorly removed [142]. Notably, the most important and abundant uremic toxins are indoxyl sulphate and p-cresyl sulphate, which are risk factors for CVDs, specifically on the pathogenesis of atherosclerosis [139].

Several reports have described the role of indoxyl sulphate in the induction of endothelial expression of adhesion molecules such as ICAM-1, as well as pro-inflammatory cytokines like nuclear factor-κB (NF-κB) and ROS production in cultured ECs [143,144]. Additionally, indoxyl sulphate is uremic toxin which induce cell senescence through p53 protein increase and ROS production [145]. These observations confirm that indoxyl sulphate produces oxidative stress and endothelial damage (dysfunction and senescence) and enhances vascular inflammation also.

Indoxyl sulphate, as well as p-cresyl sulphate, induce the release of endothelial MVs (EMVs), a marker of endothelial cell damage [146,147]. Once again, it demonstrated the new role of MVs in endothelial dysfunction during atherosclerosis progression but in CKDs patients was caused mainly by protein-bound uremic toxins (indoxyl sulphate and p-cresyl sulphate). Therefore, in the understanding of the atherosclerosis pathology, MVs represent, together with the “atherosclerosis triad” constituted by oxidative stress, endothelial dysfunction and inflammation [4], a new and essential factor in the mechanism of development and progression of atherosclerosis.

Some bone metabolism markers in uremic conditions, become imbalanced (calcium/phosphate, parathyroid hormone and vitamin D levels) inducing calcium phosphate crystals in the vasculature. The VC is a predictor of CVDs and mortality in CKDs [139]. It has also been described the aortic calcification promotion and wall thickening with indoxyl sulphate treatment in hypertensive rats [148]. Therefore, the development of new therapeutic targets that could modulate the VC associated with CVDs and/or CKDs due to molecules or structures, such as MVs, open a new field against vascular diseases, among them atherosclerotic calcification.

## 4. Microvesicles in Atherosclerosis

Recently, MVs have emerged as new regulators of biological functions in atherothrombosis [19]. MVs produce inflammatory effects, cell proliferation, thrombosis and calcification in the vasculature and appear to in human atherosclerotic plaques [7,20,21,22]. However, the possible involvement of MVs, mainly derived from smooth muscle cells, in the calcification of atherosclerotic lesions was demonstrated a long time ago [149]. The substantial participation of MVs in atherosclerosis initiation and progression is a good reason to use them as a valuable biomarker in atherosclerosis development [150] which allow MVs to be considered as pharmacological targets in this disease [21,151].

High levels of MVs have been observed in the blood of patients with atherothrombotic diseases [152] and the elevation of plasma MVs, especially those of endothelial origin, is considered a marker of vascular dysfunction [153,154]. Indeed, lymph from atherosclerotic mice displays a higher concentration of EVs [155]. Although MVs seem to be the most critical EVs in the development of AP, exosomes [156,157] and apoptotic bodies [19] have also been identified in the AP.

EVs are carries of damage-associated mediators, cytokines, autoantigens and tissue-degrading enzymes that play a critical role in inflammatory diseases [158]. EMVs generated in inflammatory process regulate vascular smooth muscle cells functions by miR-146a-5p, an important regulator of inflammation [159]. Recently, a study described MVs as a biomarker for non-invasive diagnosis and prognosis, as well as their roles as novel therapeutic targets in cardiovascular, inflammatory diseases [160]. Moreover, it has been described that changes in miRNAs EVs content rather protein or lipid, regulate inflammation associated with atherosclerosis and thrombus formation [161]. EMVs correlated with circulating endothelial markers of inflammation (soluble vascular cell adhesion molecule 1, soluble Cluster of differentiation 40 ligand; CD40L) and a prothrombotic state (von Willebrand factor; vWF) [161]. Platelet-derived MVs carried CD40L and are one of the responsible to activate endothelial cells and recruit activated platelets to endothelium damage [161].

MVs accumulate in the lipid core of the AP [153,154] and are considered as inflammatory factors in atherosclerosis [19,152,162]. However, it is essential to consider that MVs involved in AP development proceed from different cell types and influence inflammation through multiple mechanisms. APs accumulate not only large numbers of plasma MVs [152] but smooth muscle cells, fibroblasts or mesenchymal cells of the arterial wall also generate MVs involved in the formation of APs. These MVs favour local inflammation because they contain molecules that, among other effects, promote the expression of intercellular adhesion molecules, stimulate angiogenesis and local apoptosis and increase thrombus formation when AP disrupts [152].

Ordinarily, the MVs-induced clotting effect is mainly due to the exposure of phosphatidylserine in the exoplasmic face of the MVs, although MVs also carry out some cytokines and other molecules involved in cellular adhesion that also can be included in this process. The presence of intercellular adhesion molecules in the AP promotes a reduction of mobility and the hold of MVs to the AP [162,163]. MVs can also facilitate the adherence of leukocytes to ECs helping to the progress of inflammation in the AP [164].

Aside from the effect on migration and adhesion of immune cells, other inflammatory mechanisms induced by MVs have also been reported [165]. AP MVs can promote maturation and activate T-helper cells, dendritic cells and antigen presenting cells and even induce liberation of cytokines involved in the development of the inflammatory response [152]. Due to the expansion of AP being considered a chronic inflammatory process, macrophage-derived MVs have a key role in this process. They not only contain proinflammatory molecules and enhance monocyte adhesion and migration but also induce matrix degradation and plaque instability through the secretion of proteolytic enzymes [166].

A key, although heterogeneous, role in the AP development has been ascribed to miRNAs of MVs. MVs derived from platelets, ECs and macrophages contain different miRNAs that regulate inflammation in APs [161]. Macrophage MVs (MMVs) includes miRNAs (miR-146a, miR-128, miR-185, miR-365 and miR-503) whose targets are genes involved in migration and adhesion [163]. Moreover, EMVs contain miR-19b, which increases atherosclerosis progression and perivascular adipose tissue inflammation [167].

**Figure 2:**
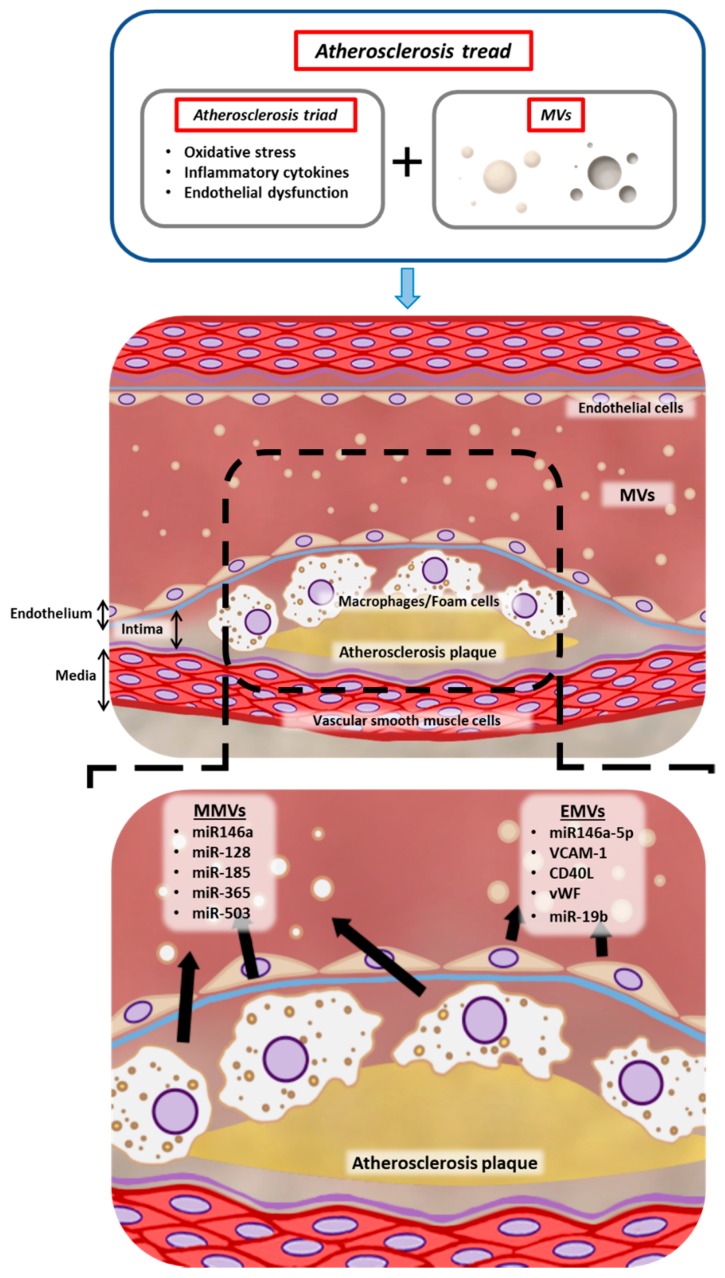
Contributors in the atherosclerosis development. Factors and microvesicles harbouring molecular mediators for the development and progression of the multistep process of atherosclerosis. Black arrows show miRNAs and factors released from macrophage MVs (MMVs) and endothelial MVs (EMVs). A dashed frame represents a zoom of the atherosclerosis plaque. Bi-directional arrow defines the different parts of vessels (endothelium, intima and media layers).

Cellular senescence increases MVs secretion [12] and this increase seems to be a general feature of cellular senescence [13]. Cellular senescence triggered by serial passaging (replicative senescence) or by cell damaged (induced senescence) such as irradiation, DNA-damaging reagents and oncogenic Ras expression enhance EVs secretion, exosomes and MVs, with a more than 10-fold increase in most cases. Several studies have revealed that senescent ECs are present in human atherosclerotic plaque [35,111,112], which suggest a specific and relevant role of senescent MVs in AP development. Due to the important role of MVs in atherosclerosis development, the authors proposed the new term “the atherosclerosis tread.” where the initial triad (oxidative stress, endothelial dysfunction and inflammation) [4] have added MVs as a contributor serving as a foundation with the other factors for the development and progression of the multistep process of atherosclerosis (Figure 2).

## 5. Microvesicles in Atherosclerotic Calcification

Involvement of cell-derived EVs in bone mineralization as well as in atherosclerotic lesion calcification has been extensively reported [7,19,25,168]. MVs have also been widely linked to the initial step and play a key role in all stages of atherosclerosis progression [19] as suggested by the term “atherosclerosis tread” (Figure 2).

Although MVs participate in the different phases of AP formation, they maintain a stronger involvement with AP calcification; in fact, for these vesicles, it has been proposed the term calcifying extracellular vesicles (CEVs) [168,169], although, with minimal differences, either ECs, smooth muscle cells or monocytes produce these CEVs [27]. Nevertheless, the role of the EVs in the regulation of the process of VC goes far beyond being a pathogenic element, since it has been demonstrated in physiological situations. Additionally, the EVs play a role in inhibiting the differentiation of the smooth muscle cells, in case they carry calcification inhibitor factors such as fetuin-A and matrix Gla (γ-carboxyglutamic acid) proteins [27] and, consequently, inhibiting the process of vesicular calcification.

Therefore, the question that arises is what makes the same structure maintain a bivalent activity due to their structural characteristics (size and membrane composition) and their contained proteins, lipids, DNA, RNA and miRNAs [170]. The answer to this question seems evident since a full panel of mediators conveyed in these CEVs seem to direct the inhibiting or activating activity of the process of calcification [27]. Different miRNAs, protein expression or the content of calcium and phosphorus in the EVs seems to play a relevant role [27,171] in this sense.

Along with this, it has also been shown that the expression of proteins, like annexins, on the surface of the CEVs plays a relevant role in the modulation of VC induced by EVs [27,49]. Advanced atherosclerotic plaques contain senescent cells, which are cell with their cell division cycles stop and release some mediators (inflammatory factors, ROS, RNS, cytokines, chemokines and matrix proteases) [140] as well as EVs. However, whether and how senescent cells contribute to atherogenesis remains unclear [67,100].

EVs contribute to the calcification process through multiple signalling pathways [172]. As above described the fetuin-A and Gla proteins are low [27] whereas others proteins that play a critical role in cell detoxification are induced, NADPH (Nicotinamide adenine dinucleotide phosphate) oxidase (intracellular calcium ion concentration) and the antioxidant superoxide dismutase-2 (SOD2) [172]. Also, in vitro, macrophages released MVs with high calcification potential. MVs expressed exosomal markers (CD9 and Tumor susceptibility gene 101 (TSG101)) and contained S100A9 (S100 calcium-binding protein A9) and annexin V [173]. Among the EVs-specific proteins, alkaline phosphatase (ALP), members of the annexin family and matrix metalloproteinase-2 (MMP-2) were found to play a prominent role in the process of matrix calcification, releasing required substrates and forming intermediates known to trigger calcium phosphate precipitation [174].

Some studies have shown the role of EVs in the different stages of atherosclerosis and subsequently in the VC [6]. Moreover, several studies have demonstrated that vascular cell-derived EVs might serve as a continuous source of damaging calcification in atherosclerotic plaques [25,26,27,169] contributing to plaque destabilization [44] and rupture, leading to CVDs.

A recent study from this current research group demonstrated that MVs from elderly subjects’ plasma or senescent ECs promoted the calcification of human aortic smooth muscle cells [49]. Additionally, with aging the number of MVs in the plasma increased and MVs released by senescent ECs contained increased amounts of calcium, calcium-binding proteins (annexin A2, annexin VI and Bone morphogenetic protein 2 (BMP2)) which are involved in VC. Along these lines, MVs could be used as markers of calcium mineral deposits that are shown like VC in atherosclerotic plaque and, therefore, MVs might offer a therapeutic target for the prevention and control of VC and associated CVDs like atherosclerosis. Likewise, MVs quantification and evaluation could be used to identify patients at risk of CVDs and/or follow the clinical course of development of VC in the atherosclerosis plaque progression.

EVs from senescent cells are released as extracellular signalling and can mediate early senescence in the cell's neighbourhood. Abbas et al. reported that MVs from patients with acute coronary syndrome developed early endothelial dysfunction, oxidative stress, premature senescence and thrombogenicity [175].

To date, the studies carried out support a role for EVs as diagnostic aid elements identifying subjects at risk of VC, mainly, MVs can be therapeutic targets in the management of these pathologies. Currently, the elimination or reduction of senescent cells, modulation of senescent factors and senescent MVs—even from the first stimulus of their formation—are a promising strategy for age-related diseases [72,99,176] and are described as senotherapies (therapies targeting senescent cells) [99].

**Figure 3:**
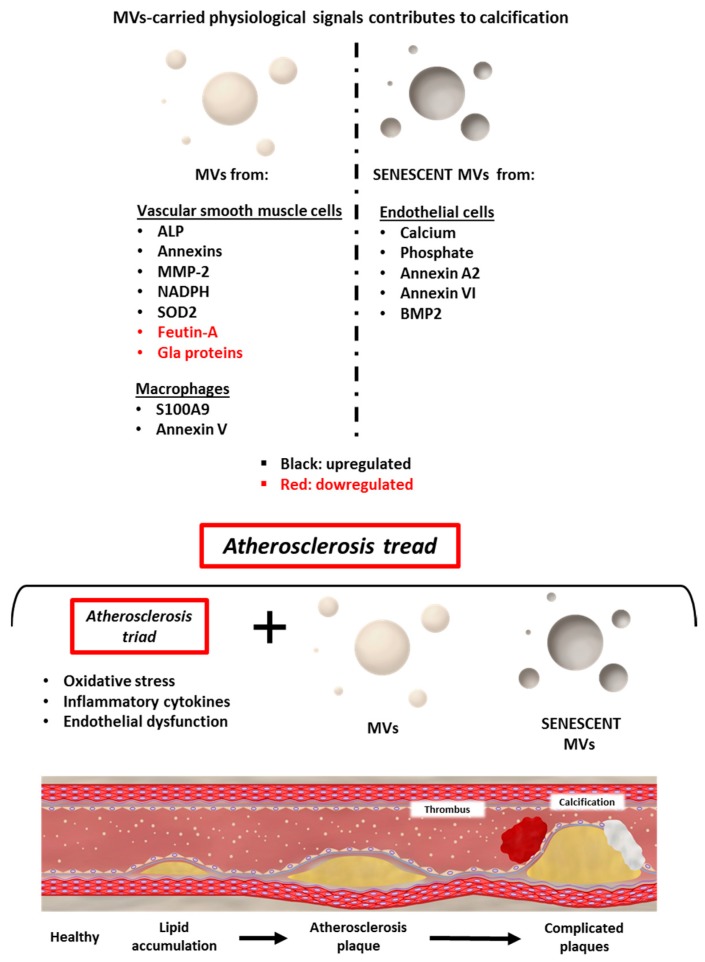
MVs in atherosclerotic calcification.

The role of the CEVs in the development of VC indicates that CEVs regulate and promote the process of VC, regardless of which cell is the origin of the EVs [168,169]. Thereby, the generation of structure-carried physiological signals (proteins and nucleic acids) that could inhibit procalcifying differentiation in smooth muscle cells and/or the activity of CEVs in the plasma of patients and at-risk subjects opens a new perspective and a new generation of targeted therapies in vascular disease associated with calcification. Figure 3 illustrates how the MVs and senescent MVs included in “the atherosclerosis tread” could be contributing to AP formation and as the last step to atherosclerotic calcification.

## 6. Conclusions

The authors have shown the role of the senescent MVs in the atherosclerotic development, specifically in the advanced plaques which, as a last step of the advanced atherosclerotic lesion, generate VC. The “atherosclerosis triad” was proposed to explain the atherosclerosis development and progression. This triad is constitutive with oxidative stress, endothelial dysfunction and inflammation. Moreover, senescence, induced by aging and/or by other associated pathologies, is a biological effect closely related with atherosclerotic lesions. MVs play a key role in the atherosclerosis development in such way that the authors have described a new term in the development of atherosclerosis named “atherosclerosis tread.” including MVs as a new factor in the progression of atherosclerosis. Finally, senescent MVs are also implicated in the complicated mechanism associated with VC. This review provides an overview of recent studies of the role of MVs, especially, senescent MVs in the VC in atherosclerosis.

## Figures and Tables

**Table 1:** Senescence cell markers.

Process	Characteristics	Markers	Regulation	References
DNA regulation	DNA replication	BrdU	↓	[74]
		^3^H-dT		
		PCNA		
		Ki-67		
	SAHFs	DAPI (DNA dye)	↑	[74,77]
	SDF	γ-H2AX	↑	[74]
		53BP1		
Lysosomal β­galactosidase activity	SA-β-gal activity	X-gal substrate	↑	[78,79]
		C_12_FDG		
Cycle control	Cell cycle arrest proteins	p16	↑	[80,81,82]
		p21		[65,83]
		p53		
		Cyclin D1		[75]
		Lamin B1	↓	[76]

BrdU, 5-bromodeoxyuridine; ^3^H-dT, ^3^H-Thymidine; PCNA, proliferating cell nuclear antigen; SA-β-gal, senescence-associated β-galactosidase; X-gal substrate, 5-bromo-4-chloro-3-indolyl-d-galactoside; C_12_FDG, 5-dodecanoylaminofluorescein di-β-d-galactopyranoside; SAHFs, senescence-associated heterochromatin foci; DAPI, 4′,6-diamidino-2-phenylindole; SDF, senescence-associated DNA damage foci; γ-H2AX, phosphorylated histone H2AX; 53BP1, p53-binding protein-1.

## Abbreviations

AP	Atherosclerotic plaque
CVDs	Cardiovascular diseases
VC	Vascular calcification
EVs	Extracellular vesicles
MVs	Microvesicles
miRNAs	MicroRNAs
ECs	Endothelial cells
HT	Hypertension
ROS	Reactive oxygen species
HBP	High blood pressure
RNS	Reactive nitrogen species
VCAM-1	Vascular cell adhesion molecule-1
ICAM-1	Intercellular adhesion molecule-1
MMPs	Metalloproteinases
NO	Nitric oxide
nLDL	Native low-density lipoproteins
oxLDL	Oxidized low-density lipoproteins
CKDs	Chronic kidney diseases
SASP	Senescence-associated secretory phenotype
SIPS	Stress-induced premature senescence
OIS	Oncogene-induced senescence
pRb	Retinoblastoma protein
SCAPs	Senescent cell anti-apoptotic pathways
SAMD	Senescence-associated mitochondrial dysfunction
SA-β-gal	Senescence-associated-β-galactosidase
BrU	5-bromodeoxyuridine
^3^H-dT	^3^H-Thymidine
PCNA	Proliferating cell nuclear antigen
SAHFs	Senescence-associated heterochromatin foci
DAPI	4′,6-diamidino-2-phenylindole
SDF	Senescence-associated DNA damage foci
γ-H2AX	phosphorylated histone H2AX
53BP1	p53-binding protein-1
X-gal substrate	5-bromo-4-chloro-3-indolyl-d-galactoside
C_12_FDG	5-Dodecanoylaminofluorescein di-β-d-galactopyranoside
Hsp70	Heat shock protein 70
MFRTA	Mitochondrial free radical theory of aging
CHD	Coronary heart disease
NEFA	Non-esterified free fatty acid
AGEs	Advanced glycosylation end products
HT	Hypertension
EUTOX	European Uremic Toxin Work Group
NF-κB	Nuclear factor-κB
BMP2	Bone morphogenetic protein 2
CEVs	Calcifying extracellular vesicles
vWF	von Willebrand factor
CD40L	Cluster of differentiation 40 ligand
Gla	γ-carboxyglutamic acid
NADPH	Nicotinamide adenine dinucleotide phosphate
SOD2	Superoxide dismutase-2
TSG101	Tumor susceptibility gene 101 protein
S100A9	S100 calcium-binding protein A9
EMVs	Endothelial microvesicles
MMVs	Macrophages microvesicles
ALP	Alkaline phosphatase
